# A Novel Postconditioning Approach Attenuates Myocardial Ischaemia-Reperfusion Injury in Rats

**DOI:** 10.31083/j.rcm2502067

**Published:** 2024-02-18

**Authors:** Lin Zhao, Yanghong Liu, Ye Chen, Zaixin Yu, Hui Luo

**Affiliations:** ^1^Department of Cardiovascular Medicine, The Third Xiangya Hospital, Central South University, 410013 Changsha, Hunan, China; ^2^Reproductive Medicine Center, The Third Xiangya Hospital, Central South University, 410013 Changsha, Hunan, China; ^3^Department of Cardiology, Xiangya Hospital, Central South University, 410013 Changsha, Hunan, China; ^4^Department of Cardiology, The First Hospital of Changsha, 410005 Changsha, Hunan, China

**Keywords:** postconditioning, ischaemia-reperfusion injury, apoptosis

## Abstract

**Background:**

Ischaemia-reperfusion injury (IRI) is the damage that occurs when blood flow is restored to a tissue or organ after a period of ischaemia. Postconditioning is a therapeutic strategy aimed at reducing the tissue damage caused by IRI. Postconditioning in rodents is a useful tool to investigate the potential mechanisms of postconditioning. Currently, there is no convenient approach for postconditioning rodents.

**Methods:**

Rats were subjected to a balloon postconditioning procedure. A balloon was used to control the flow in the vessel. This allowed for easy and precise manipulation of perfusion. Evans blue and triphenyltetrazolium chloride (TTC) double staining were used to determine the infarct size. Apoptosis in the myocardium was visualised and quantified by terminal deoxynucleotidyl transferase dUTP nick end labeling (TUNEL). Western blotting was performed to assess the expression of key apoptotic proteins, *i.e.*, B-cell lymphoma 2 (Bcl-2), Bcl-2 Associated X (Bax), and cleaved caspase-3.

**Results:**

The balloon control approach to postconditioning provided accurate control of coronary blood flow and simplified the postconditioning manipulation. Infarct size reduction was observed in IRI rats after post-conditioning. There was a decrease in cardiac apoptosis in IRI rats after conditioning, as detected by TUNEL staining. IRI rats showed increased Bcl-2 levels and decreased Bax and cleaved caspase-3 levels in the myocardium.

**Conclusions:**

Postconditioning was successfully applied in rats using this novel approach. Postconditioning with this approach reduced infarct size and apoptosis in the area at risk.

## 1. Introduction

Myocardial ischaemia-reperfusion injury (IRI) occurs when blood flow to tissues is restored after a period of ischaemia or oxygen deprivation. This is a paradoxical situation in which coronary revascularisation (reperfusion) may cause additional damage to the myocardium [1]. IRI remains a significant problem during coronary revascularisation and contributes to the morbidity and mortality of ischaemic heart disease [2].

Intensive efforts have been made to develop strategies to mitigate IRI during coronary revascularisation, including pharmacological interventions and ischaemic conditioning strategies [3]. Ischaemic conditioning has been investigated as a potential protective strategy against IRI. Although the effectiveness of ischaemic conditioning in clinical practice remains uncertain, promising preclinical studies have shown that ischaemic conditioning is cardioprotective [4]. To translate ischaemic conditioning into broader clinical applications, translational research is essential and in demand.

Ischaemic conditioning strategies include preconditioning, postconditioning, and remote ischaemic conditioning [5]. Remote ischaemic conditioning involves the application of brief episodes of ischaemia and reperfusion to a remote organ or tissue [6]. Pre- and post-conditioning refers to a series of brief episodes of ischaemia before or after the main ischaemic event [7]. Ischaemic conditioning strategies have shown potential for reducing myocardial IRI and improving outcomes.

Postconditioning refers to a series of short, intermittent periods of ischaemia and reperfusion at the onset of reperfusion (*i.e.,* immediately after blockage in the coronary artery has been removed) [8]. This is a protective strategy aimed at reducing the extent of tissue damage caused by the restoration of blood flow. Postconditioning activates several cellular signalling pathways, such as those involved in apoptosis, oxidative stress, inflammatory responses, and mitochondrial function [9]. For example, extensive studies have reported that apoptosis induced by IRI can be attenuated by post-conditioning [10]. This may be beneficial for cardiomyocyte preservation and infarct size reduction. Overall, these mechanisms may work together to limit IRI damage and promote cardiac recovery [11].

However, several aspects of these processes remain unclear. More research is needed to fully understand postconditioning and develop effective postconditioning strategies for clinical use. Easily implemented and reproducible post-conditioning strategies are essential for research in this field. Currently, the procedure for performing postconditioning in animals is complicated and difficult to control [12]. There is a need for practical and precise approaches for applying postconditioning in ischaemic animal models, which is crucial for promoting the translation of postconditioning to clinical use. Here, we present a novel and practical approach to perform precise post-conditioning in ischaemic rat hearts. The efficacy of post-conditioning was assessed in terms of infarct size, apoptosis in the area at risk and apoptosis-related protein changes.

## 2. Materials and Methods

### 2.1 Animal Grouping

All animal experiments were authorised by the institutional ethics committees of Central South University. The IRI model was established using male Sprague-Dawley rats at 6 weeks of age (body weight, 150–200 g). Animals were supplied by Changsha Tianqin Biotechnology Co., Ltd. The rats were fed standard rat chow with food and water provided *ad libitum*. They were acclimatised for one week and then randomly assigned to three groups. The number of rats in each group was as follows: N = 6 in the sham group; N = 8 in the IRI group; and N = 7 in the IRI with postconditioning (IRI + PC) group. The reporting of animal experiments followed the Animal Research: Reporting of *In Vivo* Experiments (ARRIVE) guidelines [13].

### 2.2 Anaesthesia and Incubation

The rats were not fasted prior to anaesthesia. For anaesthetic induction, rats were placed in an induction chamber, and isoflurane was delivered to the chamber at a concentration of 4% (v/v %) using an air pump and isoflurane vaporiser. Once the rat was adequately anaesthetised, confirmed by checking for loss of pedal reflex, orotracheal intubation (using a 16 gauge plastic cannula) was performed. After orotracheal intubation, the tube was connected to a small animal ventilator. The ventilation parameters were as follows: tidal volume, 2 mL; respiratory rate, 80 per minute; inspiratory: expiratory (I:E) ratio, 1:2. Isoflurane gas was continuously delivered through the tube at a concentration of approximately 2% to maintain anaesthesia during the procedure.

### 2.3 Myocardial IRI and Post-conditioning in Rats

To induce myocardial ischaemia, the left anterior descending artery (LAD) was ligated with a balloon to control infusion and occlusion. Specifically, the rats were anaesthetised using inhaled isoflurane. Appropriate anaesthetic depth was confirmed by checking the loss of the pedal reflex. The chest area of the rats was shaved and cleaned with an antiseptic solution. The rat was then placed on a heated surgical pad to maintain body temperature during the procedure. A small incision was made in the left side of the chest to expose the heart. The heart was exteriorised from the thoracic cavity. First, a silk suture was passed under the LAD. A compliance balloon dilatation catheter (2.0 mm × 15 mm) was then placed parallel to the LAD. The balloon was inflated and expanded at 12 atm. The expanded balloon was then ligated with the LAD using the suture under the LAD at the midpoint between the base and apex. Electrocardiogram (ECG) monitoring occurred throughout the procedure. The balloon was inflated at 12 atm for 30 min to induce ischaemia. At the end of the ischaemic period, rats in the IRI + PC group received post-conditioning after 30 seconds of reperfusion. Postconditioning was performed by rapidly deflating and inflating the balloon, producing intermittent periods of reperfusion and LAD reocclusion. The post-conditioning procedure consisted of four cycles of 30 seconds of occlusion reperfusion followed by 30 seconds of reperfusion reocclusion. After postconditioning, the balloon was deflated, and perfusion was performed for 10 minutes before collecting the heart tissue for morphological and molecular experiments.

### 2.4 Infarction Size Determination

At the end of reperfusion, the ascending aorta was carefully clamped using haemostatic forceps. Evan’s blue dye (2%, 2.5 mL; E2129, Sigma-Aldrich, St. Louis, MO, USA) was injected into the heart through the ascending aorta. The hearts were then collected and rapidly frozen. The hearts were then cut into 1 mm transverse slices from apex to base and stained with 1% 2,3,5-triphenyltrazolium chloride (TTC) buffer (pH 7.4 at 37 °C) for 20 minutes. The sections were fixed in 4% paraformaldehyde for 24 hours. The sections stained with Evans blue and TTC were photographed under a microscope. The perfused myocardium was stained dark blue (Evans blue). The area at risk (AAR) was not stained with Evans blue. The non-infarcted viable myocardium was stained red (TTC) and the infarcted area was left unstained (white). The infarct size (IS) and AAR were identified and quantified using the ImageJ software (FIJI 1.53c, National Institutes of Health).

### 2.5 Measurements of Apoptosis

Terminal deoxynucleotidyl transferase dUTP nick end labelling (TUNEL) staining was used to visualise apoptosis in the myocardium. Heart tissue was fixed in 4% paraformaldehyde for 24 hours at 4 °C. After fixation, the samples were processed through a series of alcohol solutions (of increasing concentrations of ethanol or methanol) for dehydration. The samples were embedded in paraffin and sectioned. The samples were then de-paraffinised and permeabilised. TUNEL staining was then performed using a TUNEL kit (C1088, Beyotime, Shanghai, China) according to the manufacturer’s instructions. Sections were counterstained with DAPI (4’,6-diamidino-2-phenylindole) to visualise the nuclei. Images were captured using a fluorescence microscope (Leica, Wetzlar, Germany). Staining and photography were performed in a blinded manner. Five random fields of the AAR near the apex were selected for each section. Images were analysed using ImageJ (FIJI 1.53c, National Institutes of Health, Bethesda, MD, USA). The apoptosis rate was expressed as the percentage of TUNEL-positive nuclei relative to the total number of nuclei.

### 2.6 Western Blotting

Proteins were extracted from the viable myocardium (the AAR) near or at the apex for western blotting experiments heart using radioimmunoprecipitation (RIPA) buffer containing protease inhibitor cocktail (P0013B, Beyotime, Shanghai, China). Protein samples were quantified using bicinchoninic acid assay. Sodium dodecyl-sulfate polyacrylamide gel electrophoresis (SDS-PAGE) gels containing 10% protein were run on polyvinylidene difluoride (PVDF) membranes (1620177, Bio-Rad, Hercules, CA, USA), and an equal volume of protein was separated. After blocking with 5% non-fat milk, the membranes were probed overnight at 4 °C with primary antibodies. Western blotting antibodies were: rabbit anti-cleaved caspase3 (Cat. No. 9664, dilution 1:1000; Cell Signalling Technology, Danvers, MA, USA), rabbit anti-Bax (Cat. No. ab32503, dilution 1:1000; Abcam, Cambridge, UK), rabbit anti-Bcl-2 (Cat. No. ab32124, dilution 1:1000; Abcam, Cambridge, UK), rabbit anti-GAPDH (Cat. No. 2118, dilution 1:5000; Cell Signalling Technology, Danvers, MA, USA). Horseradish peroxidase-conjugated goat anti-rabbit IgG (Cat. No. 9664, dilution 1:2000; Cell Signalling Technology, Danvers, MA, USA) was added to the membranes. An automated chemiluminescence imaging system (Bio-Rad, Hercules, CA, USA) was used to detect and capture the protein blots. ImageJ (FIJI 1.53c, National Institutes of Health, Bethesda, MD, USA) was used to analyse the images.

### 2.7 Statistical Analysis

Statistical analyses were conducted using *R (version 4.1.1, R Foundation, Vienna, Austria)*. The analysis of the data was done in a blinded manner. The data are presented as mean with standard deviation (SD) unless otherwise specified. Scatter plots were used to show the statistical results. Each point on the scatterplot represents one measurement and the error bars represent the SD. Student’s *t*-test was used to compare two independent groups, and one-way analysis of variance (ANOVA) was used to analyse multiple groups. Tukey’s honest significant difference method was applied for post-hoc multiple comparisons. Statistical significance was set at *p* < 0.01. The following significance codes were used: *p* < 0.01 was marked with *; *p* < 0.001 with **; *p* < 0.0001 with ***.

## 3. Results

### 3.1 A Simplified Approach to Accurately Control the Coronary Blood Flow and Perform Postconditioning

**Fig. 1.**
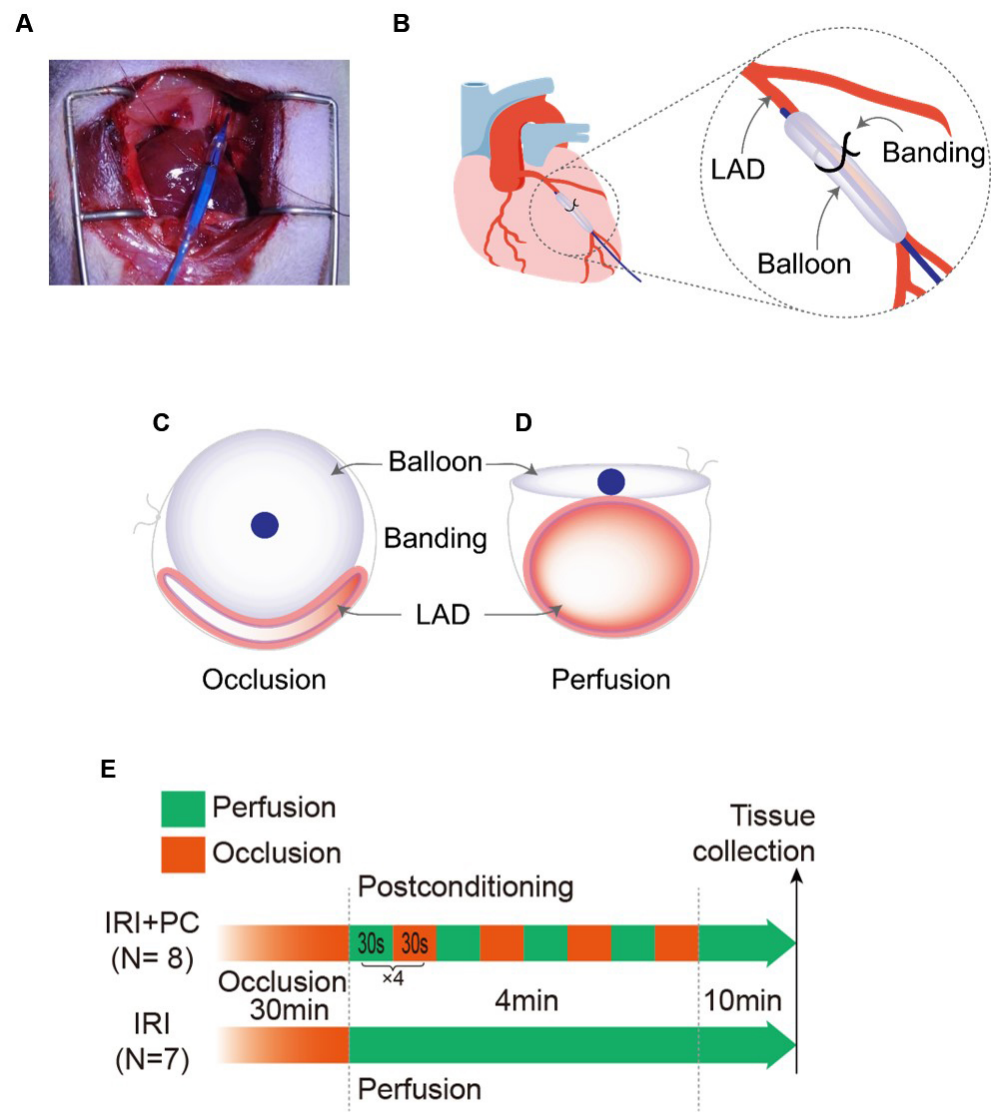
**Demonstration of implementing the novel approach of perfusion control and post-conditioning**. (A) Positioning the balloon for perfusion control and postconditioning. (B) Schematic diagram showing the position of the balloon in relation to the LAD in the novel approach. (C) The balloon inflates and compresses the blood vessel, leading to its occlusion. (D) Deflating the balloon relieves compression of the blood vessels and perfuses blood flow. (E) Diagrammatic representation of post-conditioning process. LAD, left anterior descending artery; IRI, ischaemia-reperfusion injury; PC, postconditioning.

Here, we report a novel approach for the precise control of coronary blood flow. This allows precise and rapid manipulation of the perfusion and occlusion of the coronary artery. A balloon was attached to the coronary artery, and blood flow was controlled by inflating and deflating the balloon. The placement of the balloon is shown in Fig. 1A. The balloon was placed parallel to the LAD as shown in Fig. 1B. Blood flow through the vessel is obstructed when the balloon is inflated (Fig. 1C). As the balloon was deflated, the blood flow passed through (Fig. 1D). IRI was induced by occlusion of the LAD for 30 minutes followed by reperfusion, and postconditioning was performed at the end of the occlusion (after 30-second reperfusion). Postconditioning consisted of four cycles of 30 seconds of occlusion followed by 30 seconds of reperfusion (Fig. 1E).

### 3.2 Ischemic Postconditioning Reduces IRI-induced Myocardial Infarction

**Fig. 2.**
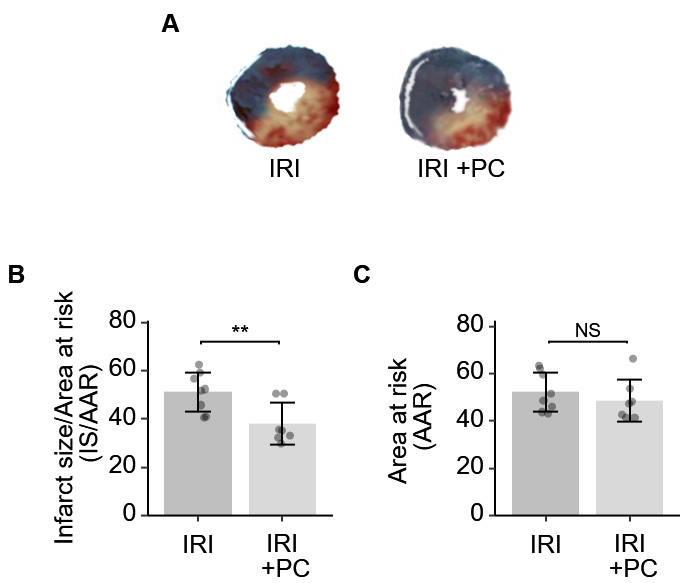
**Ischemic postconditioning decreased the infarction size in rats with IRI**. (A) Representative images of Evan’s blue and TTC stained hearts from each group. (B) Quantitative analysis of Evan’s blue and TTC dual staining suggests that post-ischemia minimizes the size of myocardial infarction. (C) The AAR, as determined by Evan’s blue, was not affected by post-conditioning. Each dot represents a measurement from one rat, and data are presented as mean ± SD. Significance codes: NS stands for not significant; ** for *p* < 0.001. TTC, 2,3,5-triphenyltrazolium chloride; AAR, area at risk; IRI, ischaemia-reperfusion injury; PC, postconditioning; IS, infarct size.

The postconditioning procedure resulted in a significant reduction in the area of infarcted myocardium in IRI, as demonstrated by the results of TTC and Evans blue staining. Representative staining images are shown in Fig. 2A. Quantitative analysis of infarct size was performed. As shown in Fig. 2B, the area of infarction was significantly reduced in IRI rats subjected to postconditioning. The AAR in IRI rats was not modified by the post-conditioning procedure (Fig. 2C).

### 3.3 Ischemic Postconditioning Reduces Myocardial IRI-induced Apoptosis in Myocardium

**Fig. 3.**
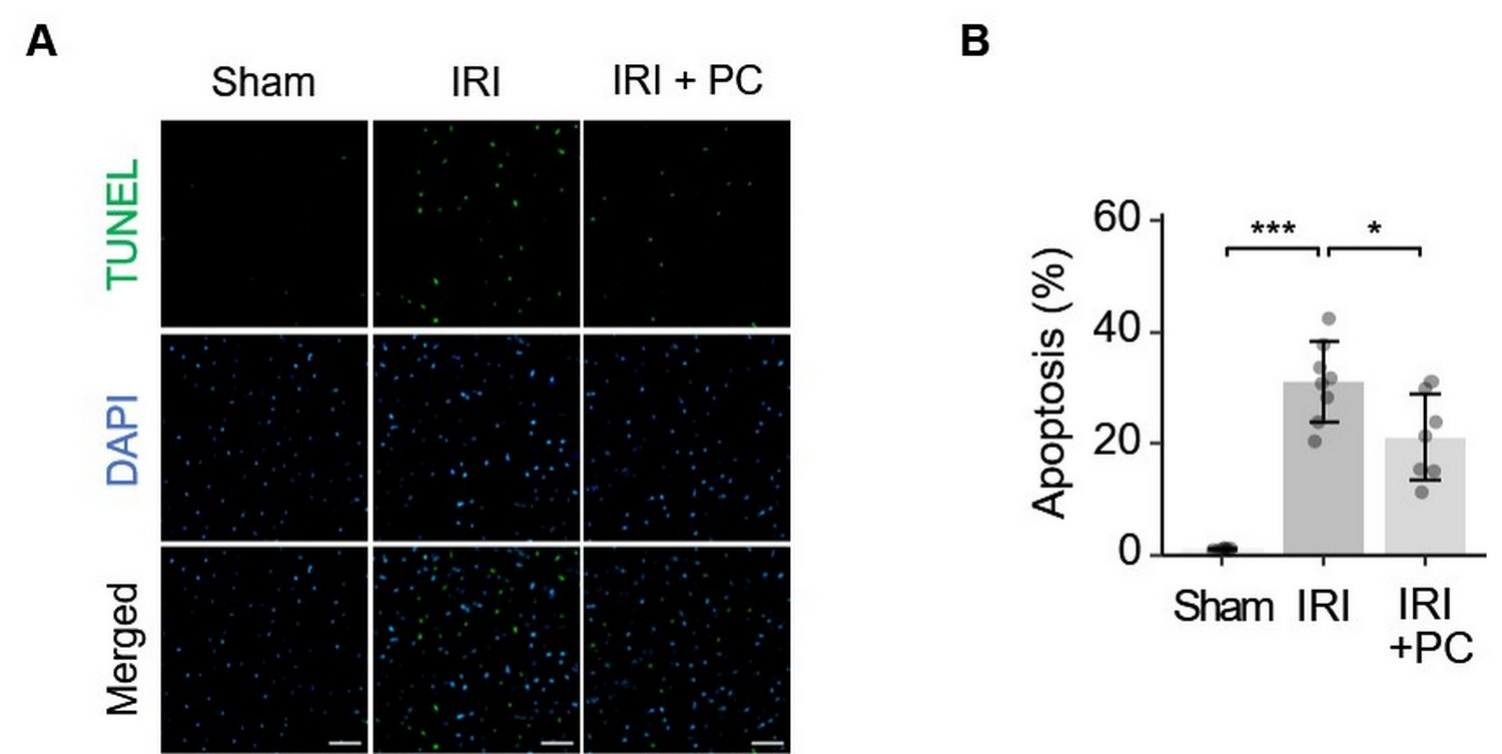
**Ischemic postconditioning reduced apoptosis in the area at risk in rats with IRI**. (A) Representative images of TUNEL staining show that postischemic reduced cardiomyocyte apoptosis in IRI; Fluorescent staining: TUNEL (green), DAPI (blue); Scale bar = 50 µm. (B) Quantitative analysis of TUNEL fluorescence images showed that IRI caused significant cardiomyocyte apoptosis in the AAR, which was attenuated by post-conditioning. Each dot represents a measurement from one rat, and data are presented as mean ± SD. Significance codes: * for *p* < 0.01, *** for *p* < 0.0001. TUNEL, terminal deoxynucleotidyl transferase dUTP nick end labelling; DAPI, 4’,6-diamidino-2-phenylindole; IRI, ischaemia-reperfusion injury; PC, postconditioning; AAR, area at risk.

Ischaemic postconditioning efficiently suppressed cardiomyocyte apoptosis in the AAR in response to myocardial IRI. This finding was based on TUNEL staining of the myocardial tissue. Representative images of TUNEL fluorescence staining are shown in Fig. 3A. Quantitative analysis of the fluorescence staining images is shown in Fig. 3B. As per the results obtained, rats with IRI presented remarkably increased apoptotic activity in cardiomyocytes in AAR, which was decreased by the postconditioning procedure.

### 3.4 Ischemic Postconditioning Lead to Changes in Apoptosis-related Proteins in the IRI Myocardium 

**Fig. 4.**
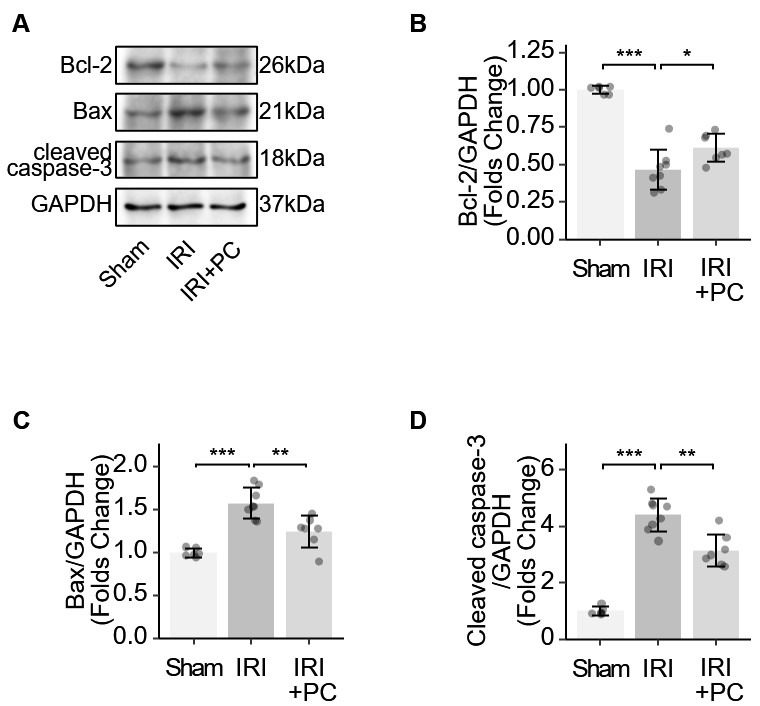
**Identification of apoptotic markers in IRI rats supports the impact of post-conditioning on apoptosis**. (A) Representative Western blotting images showing the levels of apoptosis-related proteins. (B) Quantitative analysis of western blotting of Bcl-2. (C) Quantitative analysis of western blotting of Bax. (D) Quantitative analysis of western blotting of cleaved caspase-3. Each dot represents a measurement from a single sample, and the data are presented as mean ± SD. Significance codes: * for *p* < 0.01, ** for *p* < 0.001, *** for *p* < 0.0001. Bcl-2, B-cell lymphoma; Bax, Bcl-2-associated X protein; GAPDH, glyceraldehyde 3-phosphate dehydrogenase; IRI, ischaemia-reperfusion injury; PC, postconditioning.

B-cell lymphoma 2 (Bcl-2) acts as an anti-apoptotic protein. Bcl-2-associated X protein (Bax) initiates the apoptotic cascade by activating caspase enzymes. Caspase-3 plays a key role in apoptosis and its cleavage is required to activate this process. These key markers of apoptosis were examined in IRI myocardium. Bcl-2 reduction was observed in the myocardium of IRI rats, which was reversed by the post-conditioning procedure (Fig. 4A). The myocardium of IRI rats exhibits increased levels of Bax and cleaved caspase-3. The post-conditioning procedure reduced the elevation of Bax and cleaved caspase-3 (Fig. 4B–D). The results showed that postconditioning changed these protein markers and suppressed the apoptotic mechanism in the myocardium of IRI rats.

## 4. Discussion

In the present study, we describe a novel approach for producing ischaemia and managing blood flow through rodent coronary arteries. Compared to traditional coronary ligation, the procedure is straightforward and effective, and offers quick and precise control over the period of ischaemia. Additionally, we examined the myocardial protective effects of postconditioning on IRI. Postconditioning significantly reduced the area of myocardial infarction and cardiomyocyte apoptosis in AAR. The protective effect of postconditioning on cardiomyocyte apoptosis was confirmed with regard to molecular changes. Changes in apoptotic markers suggested that postconditioning alleviated IRI-induced apoptosis.

Although preclinical studies have shown promising results regarding the cardioprotective efficacy of ischaemic conditioning, translating these findings into clinical practice has been challenging [4]. In many clinical trials, ischaemic conditioning techniques have not consistently demonstrated significant benefits in patients. Despite promising preclinical studies, the translational failure of ischaemic conditioning in clinical settings can be attributed to multiple factors. One possible reason is that animal models used in preclinical studies may not fully replicate the complex pathophysiological processes and heterogeneity of human cardiac disease [5]. Additionally, variations in study design, such as the timing, duration, and intensity of ischaemic conditioning, may contribute to the differences observed between preclinical and clinical outcomes. The translational failure of ischaemic conditioning may be due to a combination of various factors, highlighting the complexity and challenges associated with translating preclinical findings to clinical practice. Addressing the failure of ischaemic conditioning in translational research is critical as it highlights the need for better understanding and potential improvements in clinical applications. Improving preclinical *in vivo* models is helpful in the translation of ischaemic conditioning.

One basic *in vivo* research technique used to study cardiovascular diseases and evaluate potential treatments is to induce myocardial ischaemia [14]. The most commonly used rodent models for this purpose are mice and rats. Surgical procedures involve the use of either a suture or ligation device to occlude the artery, thereby inducing ischaemia in the downstream myocardium [15]. The process of suturing or deploying the ligation device can be time-consuming and therefore not suitable for achieving rapid occlusion and perfusion of the vessel. Multiple cycles of ischaemia and reperfusion are required to achieve ischaemic reperfusion [16]. A rapid and consistent method for regulating coronary blood flow is critical for performing this procedure. The need for recurrent ligation and release of the ligation on coronary arteries has made it difficult to implement postconditioning in animal models. To address this issue, we have developed this approach.

To confirm the efficacy of this method, we evaluated the infarcted area of cardiac tissue and investigated cardiac apoptosis. The findings demonstrated that this approach of post-condition deployment was effective in minimising the area of myocardial infarction following IRI and suppressing apoptosis in ARR. These results are in line with the current literature. Postconditioning has been shown to exert protective effects against apoptosis in various organs, such as the heart. By modulating multiple cellular pathways and attenuating key events in the apoptotic process, postconditioning can help preserve cell viability and improve tissue recovery following IRI [17]. The mechanisms by which postconditioning exerts its protective effects against apoptosis are complex and not fully understood. For example, postconditioning can activate pro-survival signalling pathways, such as the phosphoinositide 3-kinases/protein kinase B (PI3K/Akt) and extracellular signal-regulated protein kinases 1 and 2 (ERK1/2) pathways, which promote cell survival and inhibit apoptosis [18]. These pathways can enhance the expression of anti-apoptotic proteins and suppress their activity. Postconditioning attenuates mitochondrial dysfunction, a key event in apoptosis [19]. It can stabilise mitochondrial membrane potential, prevent the release of pro-apoptotic factors (e.g., cytochrome c) from mitochondria, and preserve ATP production [20]. Ischemia-reperfusion injury leads to the generation of reactive oxygen species (ROS) that can trigger apoptosis [21]. Postconditioning can attenuate oxidative stress by reducing ROS production and enhancing the activity of antioxidant enzymes, thereby protecting the cells from apoptosis. Ischemia-reperfusion injury triggers an inflammatory response, which can exacerbate tissue damage and promote apoptosis. Postconditioning can suppress the release of pro-inflammatory cytokines and chemokines, inhibit leukocyte infiltration, and modulate the activation of immune cells, thereby reducing inflammation-associated apoptosis [22]. Our data showed that postconditioning regulated apoptotic markers such as Bcl-2, Bax, and caspase-3. Bax and Bcl-2 are members of the Bcl-2 protein family that play critical roles in regulating apoptosis. Bax is a proapoptotic protein that promotes cell death, whereas Bcl-2 is an antiapoptotic protein that inhibits apoptosis. The balance between Bax and Bcl-2 is crucial for determining the cell fate [23]. During ischaemia-reperfusion injury, the expression and activity of Bax and Bcl-2 are altered. Ischaemia and reperfusion can increase Bax expression and decrease Bcl-2 expression, shifting the balance towards apoptosis. This dysregulation contributes to the tissue damage [24]. Postconditioning interventions have been shown to modulate the expression and activity of Bax and Bcl-2 with the aim of promoting cell survival. Postconditioning can decrease Bax expression and increase Bcl-2 expression, thereby restoring the balance between pro-apoptotic and anti-apoptotic proteins. Therefore, postconditioning can inhibit apoptosis and reduce tissue damage [25]. Caspase-3 is a key executioner caspase that is involved in the final stages of apoptosis. The activation of caspase-3 leads to the cleavage of various cellular substrates, ultimately resulting in cell death [26]. Ischemia-reperfusion injury can activate caspase-3, contributing to apoptosis and tissue damage [27]. Caspase-3 activation is associated with cleavage of specific proteins involved in cell survival and structural integrity. Postconditioning interventions can reduce caspase-3 activation, thereby inhibiting apoptosis and limiting tissue damage [28]. Our results were consistent with these observations. This also indicated that our postconditioning method was effective in significantly reducing cardiomyocyte apoptosis and inhibiting apoptosis-related pathways.

## 5. Conclusions

In conclusion, the use of this novel postconditioning method makes the implementation of postconditioning in ischemic rat myocardium easy-to-follow, convenient and effective. This approach significantly reduced myocardial infarct size in IRI and reduced cardiomyocyte apoptosis. The novel approach will further promote the research of the mechanisms of postconditioning in terms of IRI.

## Data Availability

The data that support the findings of this study are available from the corresponding author upon reasonable request.
